# Overview of Commercially Available PCR Assays for the Detection of *Aspergillus* spp. DNA in Patient Samples

**DOI:** 10.3389/fmicb.2018.00740

**Published:** 2018-04-24

**Authors:** Peter-Michael Rath, Joerg Steinmann

**Affiliations:** ^1^Institute of Medical Microbiology, Essen University Hospital, University of Duisburg-Essen, Essen, Germany; ^2^Institute of Clinical Hygiene, Medical Microbiology and Infectiology, Paracelsus Medical University, Klinikum Nuernberg, Nuremberg, Germany

**Keywords:** *Aspergillus*, aspergillosis, PCR, laboratory diagnosis, azole resistance

## Abstract

Invasive aspergillosis (IA) is a life-threatening infection in immunocompromised patients. Early diagnosis is essential to improve survival. Since the 1990s, attempts for PCR-based diagnosis of IA were made. Progress in the standardization of methods enabled the development of commercially available *Aspergillus* PCR assays in the last few years. Up to now, the clinical value of only a few commercial assays was investigated more extensively in large cohort studies. Most often, respiratory secretions such as bronchoalveolar lavage (BAL) were investigated, but some studies also included serum samples from high-risk patients. The data indicate that *Aspergillus* PCR, most likely in combination with galactomannan detection, has the potential for early and reliable diagnosis of IA including azole resistance markers. With the broad implementation of this technique in routine diagnosis and incorporation into patient care pathways, it is conceivable that an improvement in management of IA and subsequently patient outcome could occur.

## Introduction

Aspergillosis, which is defined as an infection or disease caused by fungi of the genus *Aspergillus*, mainly affects immunocompromised and critically ill patients and invasive disease is associated with high mortality. Conventional diagnosis is difficult and there is a widespread use of prophylaxis and empirical antifungal treatment in the management of invasive disease despite a relatively low incidence. Furthermore, the global emergence of infections with azole-resistant *Aspergillus fumigatus* (Steinmann et al., 2015; Verweij et al., 2016), associated with therapeutic failure, strengthened the need for rapid and reliable diagnostic methods.

The conventional diagnosis of *Aspergillus* infections is based on the presence of risk factors, radiological features, and microbiological results, i.e., histopathology and/or culture of *Aspergillus* spp. A milestone was the standardization of the classification of disease based on probability of aspergillosis [i.e., proven, probable, or possible invasive fungal disease according to the definitions of the European Organization for Research and Treatment of Cancer/Invasive Fungal Infections Cooperative Group and the National Institute of Allergy and Infectious Diseases Mycoses Study Group (EORTC/MSG; De Pauw et al., 2008)]. These criteria are the basis for determining drug efficacy, validation of diagnostic tests, and epidemiological analyses. Primarily established for the classifications of patients in clinical and laboratory studies, these definitions can also be very useful in clinical settings. However, they were established for hemato-oncological settings and modifications were necessary to fit also other patients groups like intensive care unit (ICU) patients (Meersseman et al., 2004; Blot et al., 2012). The microbiological criteria are a positive histopathology, microscopy, or culture of primarily sterile samples, defining a proven infection, and the detection of the antigens galactomannan and (1-3)-β-D-glucan in serum or other body fluids such as bronchoalveolar lavage (BAL; De Pauw et al., 2008). PCR assays were not included in 2008, but will be included in the next/current version. In recent years, *Aspergillus* PCR has been shown, in combination with other biomarkers, to be a promising tool in diagnostic algorithms as reviewed recently, for example, by White et al. (2015b), Buchheidt et al. (2017), and Lamoth and Calandra (2017).

Since the 1990s, several studies were published on in-house PCR assays to detect *Aspergillus* DNA in clinical samples. These studies showed in nearly all cases a higher sensitivity than in cultures, particularly in immunocompromised patients. However, the assays differed markedly in sample preparation, target, platforms (Buchheidt et al., 2017), and consequently in sensitivity and specificity (Arvanitis et al., 2015; White et al., 2015b). Since 2006, major progress was made in the standardization of PCR assays for the detection of *Aspergillus*-DNA in clinical samples by the European *Aspergillus* PCR Initiative (EAPCRI) Working Group of the International Society of Human and Animal Mycoses (ISHAM; White et al., 2011a). A meta-analysis reported a higher accuracy to diagnose invasive aspergillosis (IA) for those PCR studies, compliant with the EAPCRI recommendations (Arvanitis et al., 2014). The advances in the standardization of clinical criteria and laboratory procedures show that PCR is now sufficiently robust for routine *Aspergillus* diagnostics. Consequently, commercial PCR assays such as AsperGenius^®^ (PathoNostics, Maastricht, Netherlands), MycAssay *Aspergillus*^®^ (Myconostica Ltd., Cambridge, United Kingdom), MycoReal *Aspergillus*^®^ (ingenetix GmbH, Austria), RenDX Fungiplex^®^ (Renishaw Diagnostics Ltd., Glasgow, United Kingdom), MycoGenie^®^ (Ademtech, Pessac, France), LightCycler SeptiFast^®^ (Roche Molecular Diagnostics, Penzberg, Germany), GeneProof *Aspergillus* PCR^®^ (Brno, Czechia), *Aspergillus* spp. Alert Kit^®^ (Nanogen, now ELITechGroup, Turin, Italy), *Aspergillus* Real-time PCR Panel^®^ (Viracor Eurofins, Framingham, MA, United States), *A. fumigatus*^®^ Bio-Evolution (Bio-Evolution, Bry-sur-Marne, France), and Fungiplex *Aspergillus*^®^ (Bruker Daltonik GmbH, Bremen, Germany) became available in recent years. For only some of these assays data on the sensitivity and specificity for diagnosing IA are currently available. The performances of the PCR assays will be discussed in the following. In **Table 1** an overview on the performance of the assays in prospective studies is given.

**Table 1:** Overview of published prospective studies on commercial available PCR assays for *Aspergillus* detection.

Assay (Detected species)	Sample type (no. of samples/patients)	Sensitivity	Specificity	Patients	No. of patients with proven/probable IA	Reference
MycAssay	BAL (158/158)	94.1%	98.6%	Mixed	17	Torelli et al., 2011
*Aspergillus* (*Aspergillus* spp., *Penicilliurn* spp.)	Resp. samples (322/175)	86.7%	87.6%	Non-hematology	15	Guinea et al., 2013
	BAL (44/41)	80%	97.1%	Mixed	10	Orsi et al., 2015
	Serum (71/64)	46.7%	97.6%	Mixed	30 episodes	Pini et al., 2015
	Serum (358/78)	75%	Not given	Hematology	18	Oz et al., 2016
AsperGenius	BAL (201/201)	84%	80%	Hematology	52	Chong et al., 2016
(*A. fumigatus* complex, *A. terreus, Aspergillus* spp.; TR34, L98H, Y121F, T289A)	BAL (387/387)	68.4%	>92%	“at high risk”	38	Guegan et al., 2018
MycoGenie (*A. fumigatus*; TR34/L98H)	BAL (387/387)	71.1%	>92%	“at high risk”	38	Guegan et al., 2018

## MycAssay *Aspergillus^®^*

The MycAssay *Aspergillus*^®^, developed by Myconostica Ltd. (Cambridge, United Kingdom), now Microgen Bioproducts Ltd. (Camberley, United Kingdom), is a real-time PCR it for use on the Cepheid SmartCycler, Roche LightCycler 2.0, Stratagene Mx3000, AB 7500, or BioRad CFX96 platforms. The target is the 18S rDNA and the detection limit is less than 50 target copies. The assay detects different *Aspergillus* spp. including *A. fumigatus.* Torelli et al. (2011) investigated the usefulness of the MycAssay *Aspergillus^®^* in comparison with an in-house PCR and galactomannan detection in hematological *(N* = 68) and non-hematological *(N* = 90) patients using BAL samples. Most of the patients (89.9%) were neutropenic and were classified as proven, probable, or possible aspergillosis using the EORTC/MSG criteria or the modifications suggested by Meersseman et al. (2004). Two proven and 15 probable cases were found. The control group consisted of 141 patients. The sensitivity, specificity, positive predictive value (PPV), and negative predictive value (NPV) were 94.1%, 98.6%, 88.9%, and 99.3%. Nearly identical data were found by using the galactomannan assay (index cutoff of ≥1).

Guinea et al. (2013) investigated 322 respiratory samples from 175 patients without hemato-oncological diseases, of which 15 had probable IA. Sensitivity, specificity, PPV, and NPV were 86.7%, 87.6%, 34.1% and 92.2%. Culture was positive in 65 samples of 35 patients.

Orsi et al. (2012) evaluated the performance of the MycAssay *Aspergillus*^®^ in BAL samples of 20 hematological and non-hematological immunosuppressed patients, including seven suffering from IA. All patients with proven or probable aspergillosis were positive by PCR (100% sensitivity).

In a larger study (Orsi et al., 2015), the MycAssay *Aspergillus*^®^ was compared with galactomannan and culture in BAL samples from 41 patients (44 samples) at risk for infection along with assays for the detection of *Pneumocystis jirovecii*. Patients were in part hemato-oncological patients, for which the EORTC/MSG criteria were used (De Pauw et al., 2008), for patients with other underlying diseases additional host factors, such as steroid treatment, chronic obstructive pulmonary disease, lower respiratory infections, and new infiltrates were applied. Ten patients were diagnosed as probable infected with molds. Culture of BAL was positive in 6/10 patients and all had a positive galactomannan in BAL. PCR was positive in 8/10 patients, and in 1/34 of control patients. The authors calculated for the PCR assay a sensitivity of 80%, a specificity of 97.1%, a PPV of 88.9%, and a NPV of 94.3%.

White et al. (2011b) compared the sensitivity and specificity of the assay and an in-house assay using 170 serum samples from patients (104 samples of 10 patients with proven or probable IA and 66 samples from 21 patients at risk for IA). Seven of the 10 patients with proven/probable IA were positive in at least one sample. The MycAssay *Aspergillus*^®^ showed a sensitivity of 60 to 70% and a specificity of 90.5% to 100%, which was comparable to the in-house assay.

Pini et al. (2015) compared the MycAssay *Aspergillus*^®^ in serum with the galactomannan assay in 71 episodes of 64 hematological and non-hematological patients (30 episodes were classified as IA). Classification was done based on the EORTC/MSG criteria for the hematological patients and on the criteria proposed by Meersseman et al. (2004) for non-hematological patients. Sensitivity, specificity, PPV and NPV for the MycAssay *Aspergillus*^®^ were 46.7%, 97.6%, 93.3%, and 71.4%. These numbers were similar to those for the galactomannan assay. When considering hematological patients only, the sensitivity increased to 60%.

Danylo et al. (2014) compared the MycAssay *Aspergillus*^®^ in serum with galactomannan using 146 serum samples of 35 patients with hematological malignancies. Four patients suffered from a proven infection, 12 patients from a probable infection, and 13 patients were not infected. Sensitivity, specificity, PPV, and NPV were 25%, 83.3%, 39.3%, and 72%. The relative low sensitivity might be a result of a high proportion of patients under antifungal treatment in the proven/probable group (14/16 patients). The galactomannan assay showed a higher specificity (93.1%).

Another recent study also tested the MycAssay *Aspergillus*^®^ in comparison with an in-house PCR and galactomannan in 358 sera from 78 febrile neutropenic episodes in patients with IA and 83 episodes in patients with no IA (Oz et al., 2016). The topic of this study was the investigation of factors that can influence test results. The hospitalization period, duration of neutropenia, and T-cell suppression were significantly higher in the IA group. Even though there were no significant differences in test performances, the use of larger volumes (>500 μl) of sera improved the performance of the commercial PCR assay.

Taken together, the MycAssay *Aspergillus*^®^ showed a very good performance in BAL samples, especially for patients with hematological malignancies. The accuracy in the serum was not better than galactomannan.

Aquino et al. (2012) compared the performance of two commercial real-time PCR assays and galactomannan in BAL of 47 ventilated patients with chronic obstructive pulmonary disease. The included assays were the MycAssay *Aspergillus*^®^ and the *Aspergillus* spp. q-PCR Alert^®^ Kit (ELITechGroup, Turin, Italy). The *Aspergillus* spp. q-PCR kit, which is a quantitative real-time PCR with a target within the 28S rDNA, detected one of two patients with positive *Aspergillus* culture and positive galactomannan. The MycAssay *Aspergillus*^®^ was positive in 10 patients including the two with positive culture.

### AsperGenius^®^

The AsperGenius^®^ assay is a multiplex real-time PCR assay developed by PathoNostics (Maastricht, Netherlands). It runs on LightCycler 480, Rotor-Gene 6000 (Corbett) and Rotor-Gene Q (Qiagen). Two PCR assays for respiratory secretions are available, one for the detection and differentiation of *Aspergillus* spp., targeting the 28Sr DNA [AsperGenius Species multiplex^®^ with a probe for the *A. fumigatus* complex (including *A. fumigatus*, *A. lentulus*, *A. udagawae*, and *A. viridinutans*), a probe for *Aspergillus* spp. (including *A. fumigatus* complex, *A. terreus*, *A. flavus*, and *A. niger*), and one (AsperGenius Resistance multiplex^®^)] for the detection of four azole resistance markers within the Cyp51A gene of *A. fumigatus* (L98H, TR34, T289A, Y121F).

Chong et al. (2015) investigated BALs from 10 hematologic and 12 non-neutropenic patients. For neutropenic patients the EORTC/MSG criteria were used, and most of the non-neutropenic patients and some hematological patients were classified as non-classifiable. There was one proven IA case. Sensitivity, specificity, PPV, and NPV were 84.2%, 91.4%, 76.2%, 96.6%, respectively. In two cases azole-resistant strains were found.

A multicenter study from 2016 aimed to validate the assay by using stored BAL samples from patients with hematological diseases with suspected IA (Chong et al., 2016). From a total of 201 included samples, 88 were positive and 113 were negative controls. A cut-off value of <38 cycles was used. The diagnostic performance was the following: sensitivity 84%, specificity 80%, PPV 76%, and NPV 87%. In eight samples, a resistant mutation was detected. Mortality was 2.7 times higher in patients with isolates with azole-resistant mutations.

The assay was also tested by Schauwvlieghe et al. (2017) using BAL samples from 91 patients from Netherlands with positive galactomannan in BAL and suspected invasive *Aspergillus* infection. In 79% DNA of *A. fumigatus* or *Aspergillus* spp. was detected. A mutation associated with azole resistance was detected in eight cases (TR_34_/L98H) and in three patients, T289A/Y121F alterations were found.

Montesinos et al. (2017) evaluated the assay by using BAL from 100 patients including 29 patients (cystic fibrosis, lung transplant recipients) with proven or probable aspergillosis. Twenty-seven patients were positive for *Aspergillus* spp. DNA and 20 patients for *A. fumigatus* DNA. Three of them harbored mutations in the Cyp51A gene (one patient with colonization only).

In a retrospective study White et al. (2015a) investigated 124 serum samples of 49 hemato-oncological patients (14 proven/probable cases, 33 control patients). Sensitivity was 78.6% and specificity 100%. No resistance markers were found.

In a larger study, the same group (White et al., 2017) investigated 211 plasma samples from 10 patients with proven or probable IA, two possible cases and 27 controls. Sensitivity and specificity was 80% and 77.8%. If more than one positive sample was required for being relevant, the specificity increased to 100%, but sensitivity dropped to 50%.

Based on the existing studies, the AsperGenius^®^ assay had a good diagnostic utility. An innovative advantage of this of the assay is the ability to detect azole-resistant mutations.

### MycoGenie^®^

The MycoGenie^®^ assay, developed by AdemTech (Pessac, France) detects *A. fumigatus* based on the 28S rDNA, and the TR34/L98H mutation. Recommended materials are biopsies and respiratory and serum samples. The platforms are CFX96, ABI 7500, LightCycler 480, SmartCycler, MX3000, and the Rotor-Gene. Recently Dannaoui et al. (2017) validated the assay using 88 respiratory samples (59 culture positive) and 69 serum samples (from 16 patients) with proven or probable aspergillosis. Sensitivity and specificity was 92.9% and 90.1% for the respiratory samples, and 100% and 84.6%, respectively, for serum samples, when analyzing samples at the time of clinical diagnosis.

A large study by Guegan et al. (2018) from France compared the MycoGenie^®^ assay with the AspergGenius^®^ assay and two in-house assays included BAL samples from 387 hematological and non-hematological patients. One patient suffered from a proven infection, 37 patient cases were classified as probable, and 23 as possible IA. The MycoGenie^®^ assay showed a sensitivity of 53.7% in patients *(n* = 41) with hemato-oncological diseases, and a sensitivity of 75% in the 20 patients with non-hematological diseases. The AspergGenius^®^ assay showed a lower sensitivity of 41.5% in the hematological patients compared to 60% in the non-hematological patients. Not surprisingly, the sensitivity of all assays was lower in patients with antifungal prophylaxis (36–45% vs. 50–62%) or treatment (35–40% vs. 53–70%). Due to the study design, data on the specificity of the assays were not collected (analysis samples of patients with IA only). Interestingly, resistance markers were not found.

The assay was also used for molecular detection of *Aspergillus* in French patients with clinically diagnosed fungal rhinosinusitis and positive microscopy (*N* = 137), most of them with fungal ball as clinical manifestation (Morio et al., 2018). In 77.4% of the patients the assay gave positive results compared with 32.1% culture positivity. Again, no resistance markers were found.

In a recently published study, the MycoGenie^®^ assay was compared with the *A. fumigatus* Bio-Evolution^®^ assay (Bio-Evolution^®^, Bry-sur-Marne, France). The *A. fumigatus* Bio-Evolution assay uses the ITS1 region for specific detection of *A. fumigatus* DNA. In the retrospective study by Denis et al. (2018), 73 BAL samples from hematological and non-hematological patients, previously used for *P. jirovecii* PCR, were retrospectively analyzed. Thirty-one patients were classified as probable IA, 11 as probable IA, and 31 patients as control group. Both assays showed a 100% specificity and a sensitivity of 81% (*A. fumigatus* Bio-Evolution assay) and 71% (MycoGenie^®^ assay). Interestingly, antifungal treatment had no influence on sensitivities. No resistance markers were found.

In summary, the diagnostic performance of this new assay is promising; also the possibility to detected one of the most common azole resistant mutation is advantageous. More data is needed to give a final rating about the utility in the clinical routine.

### SeptiFast^®^

The SeptiFast^®^ assay is a multiplex-real-time PCR for the detection of a spectrum of bacterial and fungal pathogens in whole blood. Besides *Candida albicans*, *Candida tropicalis*, *Candida parapsilosis*, *Candida krusei*, and *Candida glabrata*, it also detects *A. fumigatus*. With respect to bacterial pathogens, the system showed a sensitivity and specificity of 68% and 86% when compared to blood culture (Dark et al., 2015).

A retrospective study by Steinmann et al. (2016) analyzed the diagnostic performance for *A. fumigatus* detection and the relevance of the assay in whole blood from critically ill ICU patients with probable or proven IA (*N* = 38) and without IA (*N* = 100). Modifications of the EORTC/MSG criteria (additional risk factor: an ICU stay longer than 4 days) were used to classify the patients. The assay showed in these patients a sensitivity of 66% and a specificity of 98%, a PPV of 93%, and an NPV of 88%. One important result was the high mortality in patients with positive PCR.

The accuracy of the SeptiFast^®^ assay for whole blood was very good. However, the disadvantages of this assay are the laborious workflow and the higher costs compared to other species-specific PCR tests.

### RenDx Fungiplex^®^

The RenDX Fungiplex^®^ assay (Renishaw Diagnostics Ltd Glasgow, United Kingdom) is based on a PCR targeting sequences of the 18S rDNA and 28S rDNA, followed by a surface-enhanced Raman scattering (SERS) for the sensitive detection of pathogen-specific oligonucleotides. It used an *Aspergillus* probe in combination with a broad-range *Saccharomycetales* probe to simultaneously detect *Aspergillus* and *Candida* spp. White et al. (2014) validated this assay, using plasma samples from 14 mostly hemato-oncological patients with proven/probable IA and 80 patients without IA. Sensitivity was 82.2% and specificity 87.5%. The RenDX Fungiplex^®^ has been reformatted to utilize real-time PCR technology. Results concerning the clinically utility of the Fungiplex *Aspergillus* PCR^®^ assay (Bruker Daltonik GmbH, Bremen, Germany) are so far not available. However, first results with spiked samples showed an excellent analytical performance in the detection of DNA of *A. fumigatus*, *Aspergillus flavus*, *Aspergillus niger*, and *Aspergillus terreus* (Green and Dougan, 2017).

## Conclusion

A number of commercial tests to detect *Aspergillus* spp. in clinical samples are available, but only a few have been currently (March 2018) validated independently from the manufacturers. Nevertheless, based on the published data so far, the mentioned assays seem to have comparable sensitivities and specificities. Overall, sensitivity is significantly lower in serum samples than in respiratory specimens. Some data indicate a lower sensitivity in patients with antifungal prophylaxis or treatment.

At the moment, the MycAssay *Aspergillus*^®^ and the AsperGenius^®^ assay can be recommended for routine PCR-detection of *Aspergillus* spp. DNA in respiratory samples. It is expectable that more PCR assays will be commercially available in the near future and thus, evaluating (multicenter) studies should also focus on the comparison of different PCR assays. Furthermore, other clinical patient specimens than serum or respiratory secretions should be included in future studies. Cerebrospinal fluid, biopsies, and pleura infusion for example are also relevant specimens for *Aspergillus* diagnostic.

## Author Contributions

P-MR and JS contributed equally by checking the literature and writing the manuscript.

## Conflict of Interest Statement

The authors declare that the research was conducted in the absence of any commercial or financial relationships that could be construed as a potential conflict of interest.
